# IL28B genotype predicts response to chronic hepatitis C triple therapy with telaprevir or boceprevir in treatment naïve and treatment-experienced patients other than prior partial- and null-responders

**DOI:** 10.1186/s40064-015-1137-x

**Published:** 2015-07-16

**Authors:** Giorgio Calisti, Amanda Tavares, Malcolm J Macartney, Adele McCormick, Wendy Labbett, Michael Jacobs, Geoffrey Dusheiko, William M Rosenberg, Tanzina Haque

**Affiliations:** Department of Virology, Royal Free London NHS Foundation Trust, Pond Street, London, NW5 4AP UK; The UCL Institute for Liver and Digestive Health/Royal Free London NHS Foundation Trust Viral Hepatitis Service, London, UK

**Keywords:** Hepatitis C, HCV, Telaprevir, Boceprevir, IL28B, Predictive value

## Abstract

Single nucleotide polymorphisms (SNPs) in the IL28B gene were shown to have limited utility in predicting response to telaprevir and boceprevir in treatment of chronic HCV infection in clinical trials. Data outside of the clinical trial setting are lacking. We assessed the value of single and combined IL28B SNPs rs12979860 and rs8099917 genotypes in predicting sustained virological response 12 weeks after cessation of triple therapy (SVR12) with telaprevir or boceprevir in a single-centre cohort of treatment-naïve and treatment-experienced patients with genotype 1 HCV mono-infection (n = 105). The overall SVR12 rate was 65.7%. By unadjusted bivariate logistic regression analysis, rs12979860-CC and rs8099917-TT were significantly associated with SVR12 in the subgroup of patients including all naïve patients and all treatment-experienced patients with the exception of partial- and null-responders to previous HCV therapy. The predictive value of rs12979860-CC was stronger than rs8099917-TT and only rs12979860-CC remained significantly predictive of treatment success when the two variants were assessed by adjusted logistic regression analysis in the whole study cohort. In patients presenting the rs12979860-CC variant, the additional determination of rs8099917 genotype had no value. IL28B rs12979860-CC remained significantly associated with SVR12 also in the multivariate analysis including the other baseline characteristics associated to SVR12 in the bivariate analysis (i.e., female gender, HCV genotype 1b, baseline viral load <800,000 IU/mL, advanced liver fibrosis and prior partial- or null-response to HCV therapy). Our study suggests that testing for the IL28B rs12979860 genotype may still be useful in predicting response to triple therapy with boceprevir and telaprevir in naïve patients and treatment-experienced patients other than partial and null-responders.

## Background

In 2009, genome-wide association studies showed that single nucleotide polymorphisms (SNPs) located upstream the gene for interleukin-28 (IL28B) on chromosome 19 were strongly associated with sustained virological response (SVR) to hepatitis C virus (HCV) treatment with pegylated interferon alpha PEG-IFN and ribavirin RBV (Ge et al. 2009; Suppiah et al. 2009; Tanaka et al. 2009). SNPs at rs12979860 and rs8099917 were identified: favourable outcome were seen with CC at rs12979860 compared to non-CC genotypes (CT or TT), and unfavourable outcome were seen with GG or GT genotypes at rs8099917. The IL28B gene encodes for a protein named interferon-lambda-3, a type III interferon (IFN). The precise molecular mechanism underlying the influence of IL28B polymorphism on response to PEG-IFN/RBV treatment for hepatitis C remains elusive. Based on the observation that patients carrying the unfavourable IL28B genotypes seem to have an inappropriately up-regulated intrahepatic expression of interferon-stimulated genes (ISGs), some authors have hypothesised that those with a favourable IL28B profile, having a lower intrahepatic ISGs expression, may be more sensitive to exogenous IFN and, thus, more likely to respond to treatment and to eradicate the infection (Honda et al. 2010; Urban et al. 2010).

Two years after the influence of IL28B SNPs was described, the first-generation protease inhibitors boceprevir and telaprevir were approved for treatment of genotype 1 chronic HCV infection in combination to PEG-IFN/RBV. These drugs substantially improved SVR rates for genotype 1 HCV in both naïve and treatment-experienced patients (Poordad et al. 2011; Jacobson et al. 2011; Bacon et al. 2011; Zeuzem et al. 2011; Pearlman 2012). The increased potency of these new directly acting agents (DAAs), has, however led some to question whether IL28B genotype testing still has any value (Jensen and Pol 2012). Retrospective analyses of phase-3 clinical trials of telaprevir and boceprevir have been conducted to evaluate the utility of IL28B genotype testing, but there are limited data outside clinical trials (Poordad et al. 2012; Pol et al. 2013; Jacobson et al. 2011).

This study assessed the value of two IL28B SNPs, rs12979860 and rs8099917, in predicting SVR to telaprevir or boceprevir triple therapy 12 weeks after treatment cessation (SVR12) in a single-centre cohort of previous HCV treatment-naïve and treatment-experienced patients treated outside of the clinical trial setting.

## Methods

### Patient selection

Patients with genotype 1 chronic HCV infection who received PEG-IFN/RBV and either telaprevir or boceprevir at The Royal Free London NHS Foundation Trust (UK) between June 2011 and December 2012 and had IL28B SNPs testing were included in the study. Prior to June 2012, patients started triple therapy through an expanded access programme (EAP) preceding approval of telaprevir and boceprevir by the National Institute for Health and Clinical Excellence (NICE). Patients co-infected with HIV or HBV and patients infected with HCV genotypes other than 1 were excluded. All patients were treated in accordance with the manufacturer’s prescribing instructions.

Institutional review board approval was not required for this study as patients were treated with approved diagnostic and therapeutic procedures according to generally accepted standards of care and all data collected retrospectively were fully de-identified.

### Data collection

Demographic, clinical and virological characteristics were collected retrospectively reviewing the clinical notes and the electronic laboratory reporting system.

Liver fibrosis was evaluated by liver biopsy performed within 24 months prior to treatment initiation in 45% of patients and by transient elastography in 25.8% of patients. In the remaining 29.2% of patients no liver biopsy or transient elastography was done because the diagnosis of liver cirrhosis was clear from routine clinical-radiological assessment. Fibrosis stage was categorised according to the METAVIR score. Where fibrosis was assessed by transient elastography the following cut-off values were used: <7.1 kilopascal (kPa) for METAVIR F0–F1, 7.1–9.4 kPa for F2, 9.5–12.4 kPa for F3 and >12.5 kPa for F4 (Castera et al. 2005). All patients diagnosed with liver cirrhosis in the routine clinical-radiological assessment were classified as METAVIR F4.

As far as previous HCV treatment is concerned, patients were considered ‘naïve’ if they had no previous treatment with PEG-IFN/RBV, ‘null-responders’ if they failed previous HCV treatment with a viral load (VL) decrease of <2 log_10_ IU/mL, ‘partial responders’ if they had a >2 log_10_ drop in VL but failed to clear the virus, ‘virological breakthrough’ if they achieved an undetectable VL initially but became viraemic again whilst on treatment, ‘relapsers’ if they achieved an undetectable HCV VL at the end of treatment (EOT) but relapsed during the subsequent 24-week follow-up and ‘interruption due to side effects (SEs)’ if discontinued prematurely treatment due to adverse reactions.

### Outcomes

SVR12, defined as undetectable HCV VL at week 12 of follow-up after end of triple therapy treatment, was the primary outcome in our intention-to-treat analysis. The secondary outcome was a shortened course of triple therapy among patients considered eligible.

### Measurements

HCV RNA was extracted from 1.5 mL of serum or plasma using the QiaSymphony platform (Qiagen, Crawley, UK) and HCV RNA VL was quantified using a validated in-house real-time PCR (RT-PCR) assay that amplifies a portion of the highly conserved 5′ untranslated region (UTR) of HCV. The assay has a lower limit of quantification of 12 IU/mL.

Human genomic DNA was extracted from plasma samples using the QiaSymphony mini DNA blood extraction kit. RT-PCR was performed for two single nucleotide polymorphisms (SNPs) within the IL28B locus (C or T for rs12979860 and G or T for rs8099917) using TaqMan allelic discrimination assay, as previously described.

### Statistical analysis

Firstly, we compared the distribution of baseline characteristics and the differences in SVR12 rates in patients receiving triple therapy through the EAP and patients treated after telaprevir and boceprevir were introduced in the UK market. Since no significant difference in SVR12 rates were observed in the two groups, we proceeded to analyse the study cohort as a whole and we evaluated the significance of associations between the study population’s baseline characteristics, including the different IL28B SNPs, and SVR12 rates to triple therapy. Patients naïve to previous HCV treatment were compared to the rest of the study cohort (all treatment-experienced patients). Each treatment-experienced subgroup (i.e., previous interruption due to SEs, viral breakthrough, relapsers, partial-responders and null-responders) was compared separately to naïve patients. Because no significant differences in outcomes between boceprevir and telaprevir groups were observed, all patients were analysed as one cohort. Two-tailed Pearson’s Chi squared, Fisher’s exact and Mann–Whitney U tests were used in the bivariate analyses. Hardy–Weinberg equilibrium of observed versus predicted IL28B genotype frequencies and the degree of linkage disequilibrium between the two SNPs were assessed by two-tailed Pearson’s Chi squared test. P values of <0.05 were considered to be statistically significant.

All factors significantly associated with SVR12 in the bivariate analyses were included in the multivariate logistic regression analyses. For the multivariate logistic regression analysis, we dichotomised previous HCV treatment categories in two groups:

1. One group including naïve patients, patients who interrupted treatment due to SEs and prior virological breakthrough and relapsers;
2. another group including prior partial and null responders.

We also dichotomised HCV genotype categories in one group including only genotype 1b and the other group comprising genotype 1a and all patients with genotype 1 where subtyping was not done.

Finally, we assessed by unadjusted and adjusted logistic regression analysis the associations between the rs12979860-CC and the rs8099917-TT genotypes and SVR12 in the two previous HCV treatment sub-groups. We also calculated sensitivity, specificity, positive predictive value (PPV) and negative predictive value (NPV) of the two favourable IL28B variants in relation to SVR12.

Data analysis was performed using Stata v12 (StataCorp LP, College Station, TX, USA).

## Results

Overall, 105 patients with HCV genotype 1 infection started triple therapy with either telaprevir or boceprevir during the study period. Around half (50.5%) of the patients in our cohort, started triple therapy through the EAP. Differences in the baseline characteristics between patients enrolled in the EAP and patients selected for triple therapy following formal launch of telaprevir and boceprevir in the UK market are summarised in Table 1. Patients treated through the EAP were older (p = 0.010), more likely to be treatment-experienced (p = 0.001) and null-responders (p = 0.038) to previous HCV therapy. A smaller proportion of patients treated through the EAP achieved SVR12 compared to patients treated outside of the EAP (60.4 vs 71.2%) but this difference was not statistically significant (p = 0.245).

**Table 1:** Baseline characteristics and SVR12 rates in patients started on treatment through the EAP and patients started on treatment outside the EAP

Variable	All patients (*n* = 105)	Treated through the EAP (*n* = 53)	Treated outside the EAP (*n* = 52)	P value
Gender, female (*n*, %)	31 (29.5)	15 (28.3)	16 (30.8)	0.782
Age, years (median, IQR)	52 (10)	53 (9)	50 (7)	0.010
Age, <52 years (*n*, %)	46 (43.8)	18 (34.0)	28 (53.8)	0.04
Ethnicity				
White/Caucasian (*n*, %)	86 (81.9)	45 (84.9)	41 (78.8)	0.298
Black (*n*, %)	6 (5.7)	3 (90.6)	3 (5.8)	0.981
Other (*n*, %)	13 (12.3)	5 (9.5)	8 (15.4)	0.355
Liver fibrosis				
Non-advanced, i.e. METAVIR F0-F2 (*n*, %)	55 (52.4)	24 (45.3)	31 (59.6)	0.141
Advanced, i.e. METAVIR F3 or F4 (*n*, %)	50 (47.6)	29 (54.7)	21 (40.4)	
Previous HCV treatment				
Naïve	29 (27.6)	6 (11.3)	23 (44.2)	0.001
Interruption due to side effects	5 (4.8)	2 (3.8)	3 (5.8)	0.631
Viral breakthrough	7 (6.7)	6 (11.3)	1 (1.9)	0.113
Relapser	33 (31.4)	18 (34.0)	15 (28.8)	0.572
Partial responder	8 (7.6)	5 (9.4)	3 (5.8)	0.479
Null responder	23 (21.9)	16 (30.2)	7 (13.5)	0.038
HCV protease inhibitor				
Boceprevir (*n*, %)	17 (16.2)	11 (20.7)	6 (11.6)	0.200
Telaprevir (*n*, %)	88 (83.8)	42 (79.3)	46 (88.4)	
HCV genotype				
1a	45 (42.9)	24 (45.3)	21 (40.4)	0.281
1b	43 (40.9)	18 (34.0)	25 (48.1)	
1 non-subtyped	17 (16.2)	11 (20.7)	6 (11.5)	0.290
HCV viral load, IU/mL (median, IQR)	1,833,451 (3,459,687)	1,893,740 (3,480,802)	1,588,705 (3,024,102)	0.423
HCV viral load, <800,000 IU/mL (*n*, %)	30 (28.5)	14 (26.4)	16 (30.8)	0.621
IL28B rs12979860 genotype				
CC (*n*, %)	23 (21.9)	8 (15.1)	15 (28.8)	0.088
CT (*n*, %)	67 (63.8)	37 (69.8)	30 (57.7)	0.196
TT (*n*, %)	15 (14.3)	8 (15.1)	7 (13.5)	0.811
IL28B rs8099917 genotype				
TT (*n*, %)	45 (42.8)	22 (41.5)	23 (44.2)	0.778
GT (*n*, %)	51 (48.6)	28 (52.8)	23 (44.2)	0.378
GG (*n*, %)	9 (8.6)	3 (5.7)	6 (11.6)	0.319
SVR12 to triple therapy	69 (65.7)	32 (60.4)	37 (71.2)	0.245

**Figure 1:**
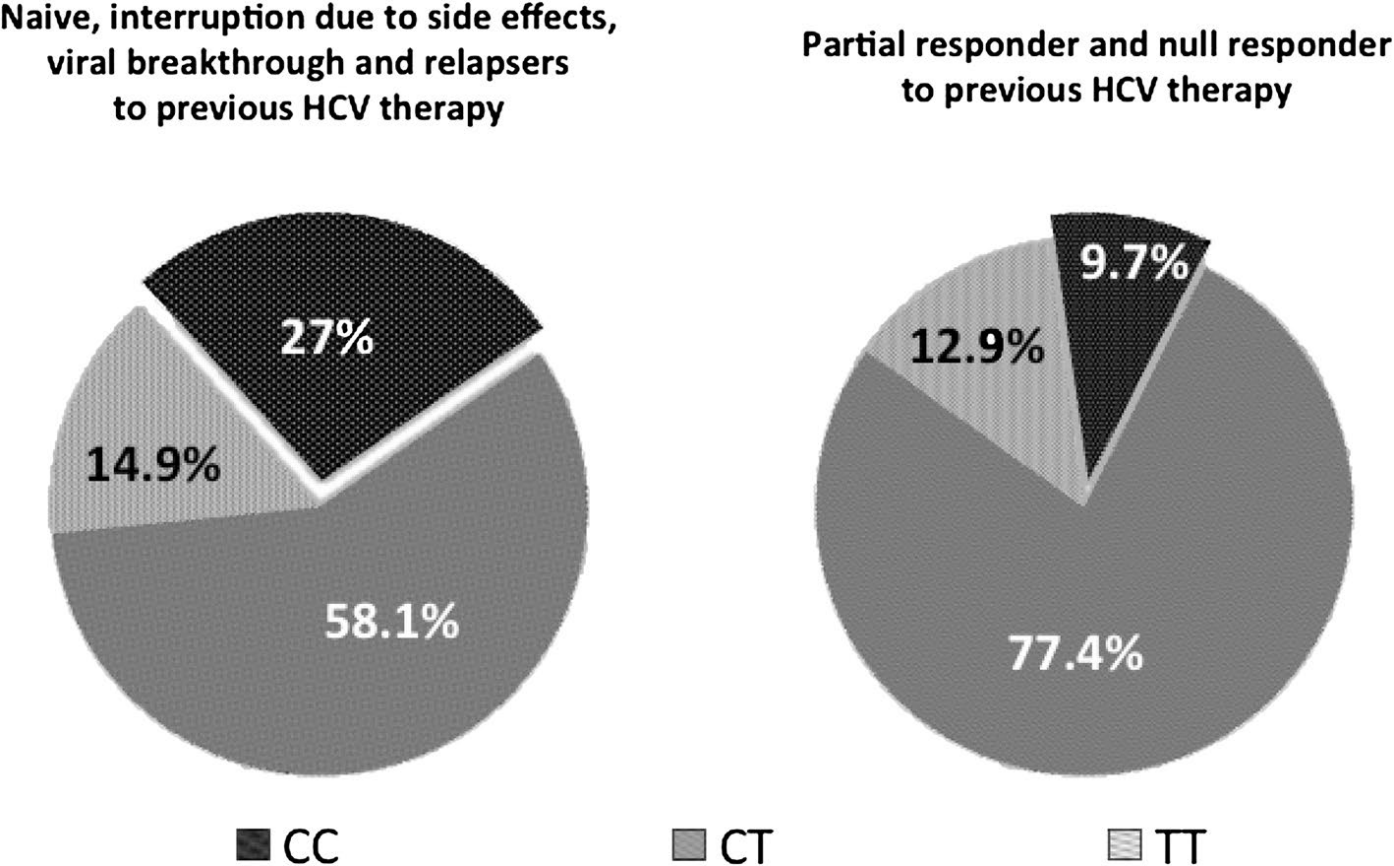
Distribution of the different IL28B rs12979860 variants according to prior hepatitis C therapy group.

Baseline characteristics, including distribution of the two IL28B SNP genotypes, and their associations with SVR 12 for the whole cohort of 105 patients are shown in Table 2. Most patients (72.4%). in the study cohort were treatment-experienced and 29.7% were partial or null responder to previous HCV therapy. Almost half (47.6%) of all patients had advanced fibrosis (METAVIR F3 or F4). Whilst the genotypic frequencies of the IL28B SNP at rs8099917 were in Hardy–Weinberg equilibrium, the genotypic frequencies of rs12979860 were not (p = 0.007), with more CT genotypes and less CC genotypes observed than expected. Strong linkage disequilibrium between IL28B s12979860 and rs8099917 was observed (p < 0.001). All 23 patients who had genotype CC at rs12979860 had also genotype TT at rs8099917, whereas only 51.1% (23/45) of patients with genotype TT at rs8099917 had genotype CC at rs12979860, the remaining 48.9% (22/45) being genotype CT. The proportion of genotype CC at rs12979860 was lower among partial- and null-responder to previous HCV treatment compared to the rest of the cohort (9.7 vs 27%, p = 0.050) (Figure 1).

**Table 2:** Baseline characteristics associated with SVR12 to triple therapy

Baseline characteristics	All patients (*n* = 105)	Patients who achieved SVR12 to triple therapy (*n* = 69)	Patients who did not achieve SVR12 to triple therapy (*n* = 36)	P value
Gender, female (*n*, %)	31 (29.5)	25 (36.2)	6 (16.6)	0.037
Age, years (median, IQR)	52 (10)	52 (16)	53 (8)	0.151
Age, <52 years (*n*, %)	46 (43.8)	33 (47.8)	13 (36.1)	0.251
Ethnicity				
White/Caucasian (*n*, %)	86 (81.9)	58 (84.1)	28 (77.8)	0.550
Black (*n*, %)	6 (5.7)	2 (2.9)	4 (11.1)	0.085
Other (*n*, %)	13 (12.3)	9 (13)	4 (11.1)	0.775
Liver fibrosis				
Non-advanced, i.e. METAVIR F0-F2 (*n*, %)	55 (52.4)	41 (59.4)	14 (38.9)	0.046
Advanced, i.e. METAVIR F3 or F4 (*n*, %)	50 (47.6)	28 (40.6)	22 (61.1)	
Previous HCV treatment				
Naïve	29 (27.6)	22 (31.9)	7 (19.4)	0.340
Interruption due to side effects	5 (4.8)	3 (4.3)	4 (11.1)	0.783
Viral breakthrough	7 (6.7)	3 (4.3)	4 (11.1)	0.393
Relapser	33 (31.4)	28 (40.6)	5 (13.9)	0.020
Partial responder	8 (7.6)	4 (5.8)	4 (11.1)	0.587
Null responder	23 (21.9)	9 (13)	14 (38.9)	0.008
Naïve, interruption due to side effects, viral breakthrough and relapsers (*n*, %)	74 (70.5)	56 (81.2)	18 (50.0)	0.001
Partial and null responders (*n*, %)	31 (29.5)	13 (18.8)	18 (50.0)	
HCV protease inhibitor				
Boceprevir (*n*, %)	17 (16.2)	13 (18.8)	4 (11.1)	0.307
Telaprevir (*n*, %)	88 (83.8)	56 (81.2)	32 (88.9)	
HCV genotype				
1a	45 (42.9)	21 (30.4)	24 (66.7)	0.001
1b	43 (40.9)	35 (50.7)	8 (22.2)	
1 non-subtyped	17 (16.2)	13 (18.8)	4 (11.1)	0.307
1a and 1 non-subtyped (*n*, %)	62 (59.1)	34 (49.3)	28 (77.8)	0.005
1b (*n*, %)	43 (40.9)	35 (50.7)	8 (22.2)	
HCV viral load, IU/mL (median, IQR)	1,833,451 (3,459,687)	1,417,630 (2,505,928)	2,115,100 (4,038,313)	0.005
HCV viral load, <800,000 IU/mL (*n*, %)	30 (28.5)	25 (36.2)	5 (13.9)	0.016
IL28B rs12979860 genotype				
CC (*n*, %)	23 (21.9)	20 (29)	3 (8.3)	0.015
CT (*n*, %)	67 (63.8)	42 (60.9)	25 (69.4)	0.385
TT (*n*, %)	15 (14.3)	7 (10.1)	8 (22.2)	0.093
IL28B rs8099917 genotype				
TT (*n*, %)	45 (42.8)	34 (49.3)	11 (30.5)	0.066
GT (*n*, %)	51 (48.6)	31 (44.9)	20 (55.6)	0.301
GG (*n*, %)	9 (8.6)	4 (5.8)	5 (13.9)	0.160
Combinations of IL28B SNPs				
rs12979860-CC and rs8099917-TT (*n*, %)	23 (21.9)	20 (29)	3 (8.3)	0.015
rs12979860-CC and rs8099917-GT (*n*, %)	0	0	0	/
rs12979860-CC and rs8099917-GG (*n*, %)	0	0	0	/
rs12979860-CT and rs8099917-TT (*n*, %)	22 (20.9)	14 (20.3)	8 (22.2)	0.514
rs12979860-CT and rs8099917-GT (*n*, %)	45 (42.9)	28 (40.6)	17 (47.2)	0.817
rs12979860-CT and rs8099917-GG (*n*, %)	0	0	0	/
rs12979860-TT and rs8099917-TT (*n*, %)	0	0	0	/
rs12979860-TT and rs8099917-GT (*n*, %)	6 (5.7)	3 (4.3)	3 (8.3)	0.404
rs12979860-TT and rs8099917-GG (*n*, %)	9 (8.6)	4 (5.8)	5 (13.9)	0.160

The overall SVR12 rate in our mixed cohort of treatment-naïve and treatment-experienced patients was 65.7%. Of the 36 patients who failed to achieve SVR12 to triple therapy, 9 patients (8.6% of the whole cohort) stopped because of side effects. Treatment was stopped due to side effects in 1 out of 17 patients started on boceprevir and 8 out of 88 patients started on telaprevir (p = 1.000). Side effects leading to treatment interruption were severe dermatitis in 2 patients, mental health problems in 2 patients, gastrointestinal intolerance in 2 patients, neutropaenic sepsis in 1 patient, severe anaemia and fatigue in 1 patient and IFN-associated retinopathy in another patient. Overall, 42.9% of patients (45/105) required either PEG-IFN or RBV dose reductions, the most common reasons being anaemia (25 patients), thrombocytopenia (11 patients) and neutropaenia (5 patients).

No significant differences in SVR12 rates were observed between patients who had their PEG-IFN or RBV dose modified compared to the rest of the study cohort (60.0 vs 70.0%, p = 0.389).

Among the baseline characteristics examined, female gender (p = 0.037), a history of relapse following previous HCV treatment (p = 0.020), HCV genotype 1b (p = 0.001), a HCV VL lower than 800,000 IU/mL (p = 0.016) and IL28B genotype CC at rs12979860 (p = 0.015) were all significantly associated with SVR12, whereas advanced liver fibrosis (p = 0.046), a history of prior null-response to PEG-IFN/RBV (p = 0.008) or the status of prior partial or null-responder to previous HCV therapy (p = 0.001) were all significantly associated to a non-response to triple therapy (Table 2). In our study, there was no added value in combining IL28B genotype CC at rs12979860 and genotype TT at rs8099917 testing compared to genotype CC at rs12979860 testing alone.

On multivariate logistic regression analysis, HCV genotype 1b (OR 3.4, 95% CI 1.20–9.51) and IL28B genotype CC at rs12979860 (OR 7.4, 95% CI 1.70–32.08) remained significantly associated with SVR12 to triple therapy, whereas advanced liver fibrosis (OR 0.3, 95% CI 0.12–0.89) and a history of previous partial or null-response to PEG-IFN/RBV treatment (OR 0.3, 95% CI 0.12–0.94) remained significantly associated with failure to achieve SVR12 to triple therapy (Table 3).

**Table 3:** Multivariate logistic regression analysis of baseline characteristics significantly associated with SVR12 to triple therapy in the bivariate analysis

Variable	Association with SVR12 to triple therapy		
	OR	95% CI	P value
Gender female	1.4	0.42–4.50	0.594
Advanced liver fibrosis (METAVIR F3 or F4)	0.3	0.12–0.89	0.029
Partial or null-responders to previous HCV therapy	0.3	0.12–0.94	0.039
HCV genotype 1b	3.4	1.20–9.51	0.021
Baseline HCV viral load <800,000 IU/mL	3.0	0.89–10.22	0.076
IL28B rs12979860-CC	7.4	1.70–32.08	0.007

*OR* odds ratio, *CI* confidence intervals.

In the unadjusted logistic regression subgroup analysis according to previous HCV treatment history, both IL28B 12979860-CC genotype and rs8099917-TT genotype were significantly associated with SVR12 in the group including patients naïve, patients who interrupted treatment due to SEs, patients with a history of viral breakthrough or relapse (OR 7.3 and 4.34, respectively) (Table 4). No significant association was found between any of the SNPs and SVR12 in the subgroup including partial and null responders to previous HCV therapy.

**Table 4:** Unadjusted and adjusted logistic regression analysis of the associations between IL28B rs12979860-CC and rs8099917-TT and SVR12 to triple therapy according to previous HCV treatment group

	All patients (105 patients)	Naïve, interruption due to SEs, viral breakthrough and relapsers (74 patients)	Partial and null reponders (31 patients)
SVR12 (95% CI)	65.7% (56.6–74.8)	75.7% (65.9–85.5)	41.9% (24.5–59.3)
IL28B rs12979860-CC			
Frequency (95% CI)	21.9% (14.0–29.8)	27% (16.9–37.1)	9.7% (0.0–20.1)
Unadjusted OR (95% CI)	4.4 (1.23–16.33)	8.7 (1.08–70.67)	0.67 (0.05–8.24)
Adjusted OR (95% CI)	3.97 (1.07–14.72)	7.3 (0.88–61.18)	0.59 (0.47–7.43)
Sensitivity (95% CI)	29.0% (19.0–41.3)	33.9% (22.2–47.9)	7.7% (0.4–37.9)
Specificity (95% CI)	91.7% (76.4–97.8)	94.4% (70.6–99.7)	88.9% (63.9–98.1)
PPV (95% CI)	87.0% (65.3–96.6)	95.0% (73.1–99.7)	33.3% (1.8–87.5)
NPV (95% CI)	40.2% (29.7–51.7)	31.5% (19.9–45.7)	57.1% (37.4–75.0)
IL28B rs8099917-TT			
Frequency (95% CI)	42.8% (33.4–52.4)	47.3% (35.9–58.7)	32.3% (15.8–48.8)
Unadjusted OR (95% CI)	2.20 (0.94–5.17)	4.3 (1.27–14.84)	0.47 (0.10–2.34)
Adjusted OR (95% CI)	0.52 (0.17–14.72)	0.46 (0.12–1.82)	0.39 (0.04–4.35)
Sensitivity (95% CI)	49.3% (37.1–61.5)	55.4% (41.6–68.4)	23.1% (6.2–54.0)
Specificity (95% CI)	69.4% (51.7–83.1)	77.8% (51.9–92.6)	61.1% (36.1–81.7)
PPV (95% CI)	75.6% (60.1–86.6)	88.6% (72.3–96.3)	30.0% (8.1–64.6)
NPV (95% CI)	41.7% (29.3–55.1)	35.9% (21.7–52.8)	52.4% (30.3–73.6)

Sensitivity, specificity, PPV, NPV of the two favourable IL28B variants in relation to SVR12.

*OR* odds ratio, *CI* confidence intervals, *PPV* positive predictive value, *NPV* negative predictive value.

In the adjusted regression analysis including both favourable SNPs, IL28B 12979860-CC maintained is significant association with SVR12 in the whole cohort and a trend towards significance was observed also in the subgroup of patients naïve, interruption due to SEs, virological breakthrough and relapsers, whereas the association between rs8099917-TT and SVR12 disappeared in all treatment groups (Table 4).

PPV of IL28B genotype CC at rs12979860 in relation to the likelihood of achieving SVR12 was 87% (95% CI 65.3–96.6%) in the whole study cohort, 95% (95% CI 73.1–99.7%) in the group including patients naïve, patients who interrupted treatment due to SEs and patients with a history of viral breakthrough during treatment or relapse after EOT. PPV of genotype rs8099917-TT in relation to SVR12 was 75.6% (95% CI 60.1–86.6) in the whole study cohort and 88.6% (95% CI 72.3–96.3) in the group including patients naïve, patients who interrupted treatment due to side effects and patients with a history of viral breakthrough during treatment or relapse after EOT.

Five out of 7 and 25 out of 34 patients considered eligible at baseline received a shortened course of therapy with boceprevir and telaprevir, respectively. Overall duration of triple therapy could be shortened in 73.2% of patients. None of the baseline characteristics, including IL28B genotype CC at rs12979860, was significantly associated with the likelihood of receiving a shortened course of triple therapy.

## Conclusions

There is a lack of published data on the utility of IL28B genotyping in predicting SVR to triple therapy with telaprevir and boceprevir. In our cohort, IL28B genotype CC at rs12979860 and genotype TT at rs8099917 were significantly associated with SVR12 and predicted successful virological cure following triple therapy in the subgroup of patients comprising both patients naïve to previous therapy and patients who failed prior treatment but showed the ability to respond to PEG-IFN/RBV (i.e., previous virological breakthrough and relapsers).

Poordad et al. (2012) reviewed the factors associated with SVR in the boceprevir studies SPRINT-2 on treatment-naïve patients and RESPOND-2, on treatment-experienced patients. The CC genotype at rs12979860 was independently associated with SVR in SPRINT-2 but not in RESPOND-2. In both studies, the rs12979860-CC genotype was associated strongly with a good response to interferon at week 4 (>1 log_10_ VL decline after the lead-in phase) and could therefore be used to identify patients eligible for shorter treatment duration. This association, however, disappeared in the multivariate regression analysis. In the ADVANCE study, which evaluated telaprevir for naïve patients, rs12979860-CC genotype patients were more likely to be eligible for shortened duration of treatment and achieve an eRVR (Jacobson et al. 2011). Finally, in treatment-failure patients treated with telaprevir-based therapy (REALIZE study), IL28B did not significantly affect SVR in the multivariate analysis (Pol et al. 2013).

In our study, the predictive value of both IL28B polymorphisms in relation to the likelihood of achieving SVR12 to triple therapy was seen only in the sub-group including the patients with favourable prior HCV treatment history (i.e., naïve, patients who interrupted treatment due to SE, patients with a history of virological breakthrough during treatment or relapse after EOT). These findings are comparable with the results of the clinical trials, which reported a significant effect of IL28B genotype only in treatment naïve individuals (Poordad et al. 2012; Jacobson et al. 2011).

In this study, rs12979860-CC was a stronger predictor of SVR12 than rs8099917-TT. In patients presenting the rs12979860-CC, the additional determination of rs8099917 genotype had no value. When the two variants were compared directly by multivariate logistic regression analysis, only rs12979860-CC remained significantly associated to treatment success. Our results are in line with several larger studies conducted in patients treated with the combination PEG-IFN/RBV (Bochud et al. 2011; Fischer et al. 2012).

Because around half of our study population was started on treatment through the EAP, the results of this study may not be entirely generalizable to the “real-world”. Although patients enrolled through the EAP may have been more difficult to cure because older and more likely to be treatment-experienced and null-responder to previous HCV therapy, we cannot exclude a selection bias whereby the treating physicians may have preferentially enrolled in the EAP the most motivated patients. This could explain why, even in this subgroup of patients where over 90% of subjects were treatment-experienced and the majority had advanced liver fibrosis, a SVR12 rate of 60.4% was observed. This is higher than the cure rate reported by the two largest “real-world” case series of patients treated with telaprevir- or boceprevir-based triple therapy published to date: Ioannou et al. (2014) analysis of the Veterans Administration (VA) healthcare system cohort and Price et al. (2014) analysis of Northern California Kaiser Permanente (KPNC) cohort. Since compliance was not consistently noted for all patients and throughout the study period, we are unable to prove the link between this hypothetical selection bias and the increased SVR12 rate. Other differences in cohort composition could explain our greater SVR12 rate. The VA cohort had a greater proportion of men (96 vs 70.5%) and a smaller proportion of patients infected with HCV genotype 1b (21.3 vs 48.9%, considering only patients who had genotype 1 subtyping performed) (Ioannou et al. 2014). In the KPNC cohort HCV genotype 1b was less frequent than in our study population (33.7 vs 48.9%, considering only patients who had genotype 1 subtyping performed) and the proportion of black individuals was greater (15 vs 5.7%) (Price et al. 2014). IL28B genotype composition was not reported in the KPNC study, however, because the CC profile at rs12979860 is less common among black patients, it is possible that a smaller proportion of patients had the most favourable profile (Clark et al. 2011).

In summary, our study suggests that, outside the clinical trials setting, IL28B testing genotype CC at rs12979860 remains predictive of SVR12 to telaprevir- and boceprevir-based triple therapy. Similar to the retrospective analyses of phase-3 clinical trials, the predictive value of rs12979860-CC was observed only in patients in the most favourable previous HCV treatment categories, such as naïve patients, subjects that interrupted PEG-IFN/RBV therapy due to adverse events, prior viral breakthrough and relapsers. More efficacious and better-tolerated DAAs are now available. Although preliminary data suggest that patients with a favourable IL28B genotype seem to have slightly better chances of cure even with some interferon-free combinations, the influence of IL28B is likely to fade away when SVR rates approaches >90% in all patients (Holmes et al. 2012). In countries where this new wave of DAAs is already replacing telaprevir and boceprevir, testing for IL28B may be considered futile. However, in those settings where the use of the newer DAAs will be restrained for some time due to limited economic resources, testing for IL28B genotype may still be used, in addition to other baseline predictors, to identify patients that can be successfully treated with the telaprevir- and boceprevir-based treatment regimens.
